# hCoronavirusesDB: an integrated bioinformatics resource for human coronaviruses

**DOI:** 10.1093/database/baac017

**Published:** 2022-03-26

**Authors:** Iman Almansour, Imane Boudellioua

**Affiliations:** Department of Epidemic Diseases Research, Institute for Research and Medical Consultations (IRMC), Imam Abdulrahman Bin Faisal University, P.O. Box 1982, Dammam 31441, Saudi Arabia; Information and Computer Science Department, King Fahd University of Petroleum and Minerals, P.O. Box 2205, Dhahran 31261, Saudi Arabia

## Abstract

In the twenty-first century, three new human coronaviruses have been identified with known zoonotic origins: severe acute respiratory syndrome coronavirus (SARS-CoV), SARS-CoV-2, and Middle East respiratory syndrome coronavirus (MERS-CoV). SARS-CoV-2 was identified in November 2019 and is associated with an ongoing pandemic. Molecular surveillance and monitoring studies are essential for containing viral outbreaks, epidemics, and pandemics. In addition, the development and deployment of bioinformatics resources for highly pathogenic human coronaviruses are crucial for understanding the genetic and immunogenic landscape associated with these viruses. Here, we introduce an open-access, integrated resource for SARS-CoV, SARS-CoV-2, and MERS-CoV: the Human Coronaviruses Database and Analysis Resource (hCoronavirusesDB; http://hcoronaviruses.net/), which include nucleotide and protein sequence data obtained for these viruses. The database also offers a user-friendly search interface coupled with bioinformatics analytics and visualization tools. In addition, hCoronavirusesDB contains curated, experimentally validated B cell and T cell epitope data for these viruses. This resource can assist with the molecular surveillance necessary to trace virus circulation and contribute to microevolutionary studies. This application can also serve as a valuable resource for the development of rationally designed pan-coronavirus diagnostic tools, vaccines, and therapeutic agents.

Database URL:http://hcoronaviruses.net/

## Introduction

Severe acute respiratory syndrome coronavirus (SARS-CoV), SARS-CoV-2, and Middle East respiratory syndrome coronavirus (MERS-CoV) are three pathogenic human coronaviruses (CoVs) (1, 2). SARS-CoV was discovered in 2002 and was associated with the 2002–2003 outbreak of SARS. MERS-CoV was discovered in 2012 and was associated with MERS outbreaks in the Middle East. The most recently identified human coronavirus, SARS-CoV-2, was discovered in 2019 and is associated with an ongoing COVID-19 pandemic (2, 3). All human CoVs are believed to be zoonotic in origin, and bats are known to serve as a natural CoV reservoir. Civet cats were identified as potential intermediate hosts for SARS-CoV, whereas camels were identified as the potential intermediate hosts for MERS-CoV (2, 3). Although SARS-CoV-2 is known to be less lethal than either SARS-CoV or MERS-CoV, nearly 20% of infected individuals develop severe respiratory symptoms characterized by interstitial pneumonia and acute respiratory syndrome. Older adults and individuals with underlying medical conditions are at particular risk for developing severe respiratory symptoms (3).

CoVs are enveloped, positive-sense, single-stranded RNA viruses and are classified genotypically and serologically into four subfamilies: alpha, beta, gamma, and delta. Human CoVs belong to the alpha and beta groups, and SARS-CoV, SAR-CoV-2, and MERS-CoV are all beta CoVs (4, 5). Genome-wide bioinformatics studies have revealed that SARS-CoV-2 and SARS-CoV share nearly 80% similarity, whereas SARS-CoV-2 and MERS-CoV share nearly 50% similarity (6–8). The high sequence identity shared between SARS-CoV-2 and SARS-CoV (nearly 95%) at ORF1ab indicates that SARS-CoV-2 belongs to the Sarbecovirus subgenus of the beta CoV group (9, 10).

Virus surveillance, coupled with viral genome sequencing, has become an important and widely used tool. The rapid increase in sequence data can be of great value for sequence-informed virus surveillance and microevolutionary studies. GISAID (https://www.gisaid.org/) and Next Strain (https://nextstrain.org/) provide valuable gene and protein sequences for SARS-CoV-2 (11, 12). These databases, however, do not integrate information for all three highly pathogenic human coronaviruses (SARS-CoV, MERS-CoV, and SARS-CoV-2). In addition, the surveillance of CoVs in non-human hosts can reveal critical mutations with the potential to develop into future outbreaks. Furthermore, the availability of experimentally characterized B cell and T cell epitope data can assist with monitoring the immune responses to viruses. Identifying unique and variable epitope regions contributes to understanding the landscape of epitope regions within human CoVs, which can have implications for the rational design of vaccines and therapeutic agents and the development of improved diagnostic tests.

To meet the need for integrated bioinformatics resources associated with human CoVs, we present the Human Coronaviruses Database and Analysis Resource (hCoronavirusesDB), which has the following features:

1. A comprehensive searchable database for CoVs that integrates existing genomics and proteomics data available for SARS-CoV, SARS-CoV-2, and MERS-CoV and includes retrieval options that can be accessed through a user-friendly interface, allowing for customized data searches based on gene product, host, and year.
2. A customized suite of analytical and visualization tools, including standalone multiple sequence alignment, sequence viewer, and geographic map distribution.
3. A catalogue of experimentally verified B cell and T cell epitopes for SARS-CoV, SARS-CoV-2, and MERS-CoV.
4. A basic local alignment search tool (BLAST) for hCoronavirusesDB.

## Materials and methods

### Data collection and processing

#### Sequence data

**Figure 1.**
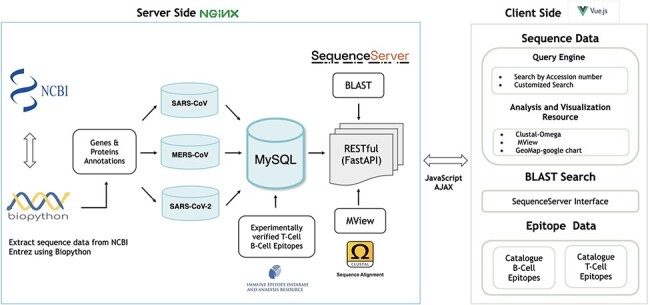
Architecture of the hCoronavirsesDB bioinformatics resource.

The gene and protein sequences for SARS-CoV, SARS-CoV-2, and MERS-CoV were retrieved from the National Center for Biotechnology Information (NCBI; https://www.ncbi.nlm.nih.gov). The NCBI Entrez online query system was utilized to access the gene and protein sequences from the NCBI Nucleotide and Protein databases, respectively, using the Biopython (13) Entrez module (Figure 1). These records were retrieved using Entrez e*search* function from Biopython for each organism with *publication date* filter up to February 27, 2021. Records representing complete genomes were dissected to extract individual gene and protein sequences, along with their respective annotations.

**Figure 2.**
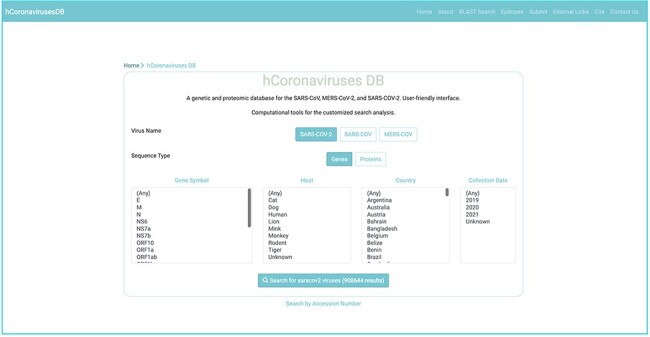
Nucleotide and protein sequences search interface for the SARS-CoV, SARs-CoV-2, and MERS-CoV.

**Figure 3.**
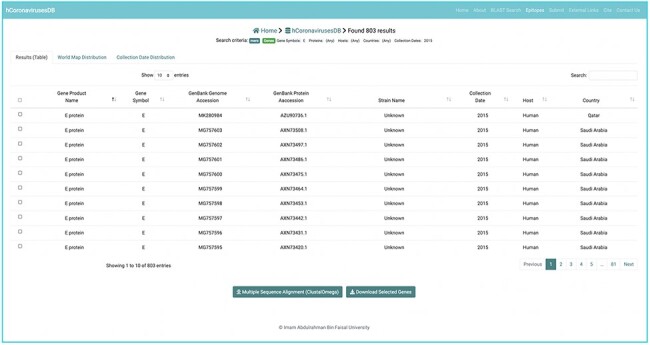
Sequence data search output.

To ensure consistent sequence data representation, the following policies have been applied. Records lacking annotations both of gene symbol and the product name were omitted. For records annotations for either the gene -name or the product name, the missing annotation was inferred based on a curated mapping table between gene names and their corresponding product name, and vice versa (Supplementary Table S1). Due to the diversity in host annotations for sequence records, host annotations were converted to a unified representation using the name of the host species (Supplementary Table S1). For example, a host annotation with the scientific name *Ia io* was converted to the common name *Bat*. (Supplementary Table S1). These unified host name representations are used to facilitate the search and retrieval of sequences of interest. However, the original host names, as provided in NCBI database can be found as part of the annotations in each record’s fasta file. Overall, every obtained gene and protein sequence record included the following annotations: gene symbol, GenBank nucleotide accession number, gene product name, GenBank protein accession number, collection date, release date, original host organism name, mapped host organism name, isolate name, country, organism name, strain name, and isolation source, and nucleotide or amino acid sequence. Any missing annotations (except for the gene symbol and product name) were labelled as *unknown* (Figures 2 and 3). As of 27 February, 2021, a total of 907 gene sequences and 896 protein sequences were obtained for SARS-CoV; 5548 gene and protein sequences were obtained for MERS-CoV; and 908 644 gene sequences and 908 158 protein sequences were obtained for SARS-CoV-2, all of which are currently available at hCoronavirusesDB.

The sanitized, processed sequence data were stored in the MySQL Relational Database Management System (https://www.mysql.com/), which interfaces with a FastAPI Python library (https://fastapi.tiangolo.com/features/; Figure 1). In addition, the sequence data were fully integrated with a set of analytical tools to assist researchers with identifying sequence mutations and performing geographic distribution analyses (Figure 1).

#### Epitope data

An epitope is defined as a group of amino acids within a protein antigen that interacts with antibodies or T cell receptors and subsequently activates the immune response. B cell epitopes are further classified as linear or discontinuous based on their structures. Linear epitopes, also known as continuous epitopes, are amino acids that appear sequentially within the primary protein sequence. Three of the four proteins in CoVs are exposed to the surface: Spike (S), nucleocapsid (N), and envelope (E) proteins (Long, 2020).

**Figure 4.**
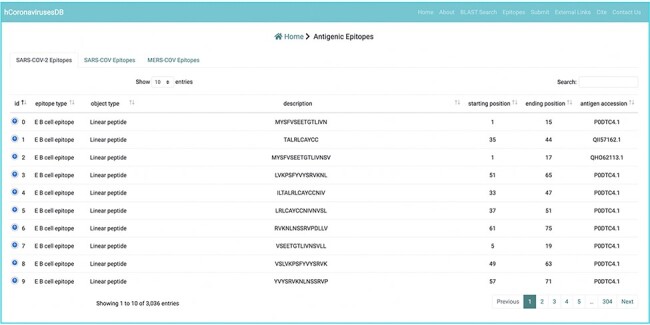
Example of an experimentally verified B cell and T cell epitope data in the hCoronavirusesDB.

Experimentally verified B cell epitope data were curated from the immune epitope database (14) (IEDB; accessed 20 October, 2021; http://www.iedb.org). We primarily focused on the S, M, N, and E CoV proteins, which are the typical targets of developed immunity against these viruses. Inferred epitope data were further subcategorized according to epitope ID, object type, description, starting position, ending position, antigen accession, organism name, and parent organism (Figure 4). The numbers of retrieved B cell epitopes for individual SARS-CoV, MERS-CoV, and SARS-CoV-2 proteins. In parallel, we sought to retrieve experimentally verified T cell epitopes from IEDB and the numbers of retrieved T cell epitopes for individual SARS-CoV, MERS-CoV, and SARS-CoV-2 proteins were included in the hcoronavirusesDB.

### Sequence alignment tools

To facilitate the study of closely related genes and proteins identified in SARS-CoV, MERS-CoV, and SARS-CoV-2, based on both functions and evolutionary relationships, we integrated into the hCoronavirusesDB two widely adopted sequence alignment methods: Clusta-Omega and BLAST.

### Clustal Omega

**Figure 5.**
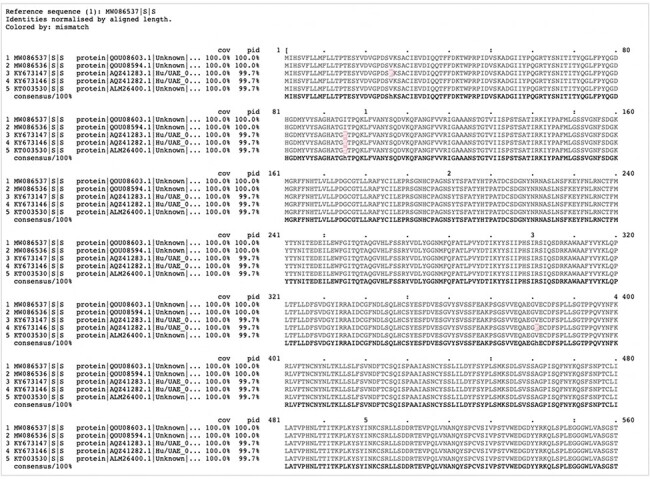
Clustal-Omega alignment of a customized search and visualized by MView.

Clustal Omega (15, 16) is the latest version in the Clustal series of algorithms for performing multiple sequence alignments. Clustal Omega is a command-line tool that offers major enhancements over previous Clustal versions in terms of scalability and accuracy, as measured across several benchmarks. The tool was integrated into hCoronavirusesDB as an analysis resource, along with the MView multiple sequence alignment viewer (17), to enable the extraction and reformatting of the resulting multiple sequence alignments into HTML markups for web page display. To obtain and visualize the alignment, the user is required to select at least two gene or protein sequences (Figure 5).

Optionally, the user can specify the reference sequence among the selected sequences, as well as the consensus sequence in the resulting alignment. The threshold for sequence similarity in MView was set to 100% (Figure 5).

### The basic local alignment search tool (BLAST)

**Figure 6.**
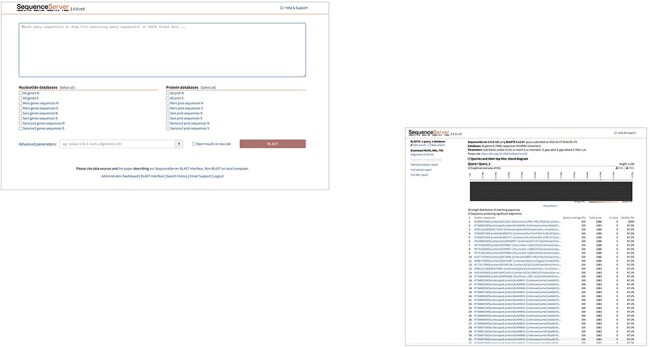
Illustration of Geo-Map of customized sequence search.

To enable the study of closely related genes and proteins identified in SARS-CoV, SARS-CoV-2, and MERS-CoV, in terms of their distributions and evolutionary relationships, sequence alignment tools were integrated into hCoronavirusesDB. BLAST (18) is an algorithm for comparing a query gene or protein sequence with a library of gene or protein sequences to discover and assess similarities based on specified thresholds and parameters. SequenceServer hosted cloud service (19) was used to establish a BLAST+ server in the database, to which users can upload query sequences in FASTA format for alignment with various libraries (Figure 6). Within the scope of this database, a total of 16 BLAST search libraries were constructed using the makeblastdb application, which is provided with the BLAST+ package (Figure 6). These libraries correspond to the gene and protein sequences available within hCoronavirusesDB for specific subset of genes of interest, namely N and S genes and their corresponding protein sequences. To generate nucleotide libraries, an individual library was constructed for either S or N genes representing one of the following viruses: SARS-CoV, SARS-CoV-2, and MERS-CoV. In addition, a combined library was constructed for either S or N gene sequences representing all three viruses. Likewise, six individual libraries were constructed representing the associated protein sequences corresponding to either S or N proteins of SARS-CoV, SARS-CoV-2, and MERS-CoV, in addition to a combined library merging S or N protein sequences for all three viruses. A customized BLAST search can be applied, and advanced parameters can be established to perform search and query filtering (Figure 6).

### Geo map

**Figure 7.**
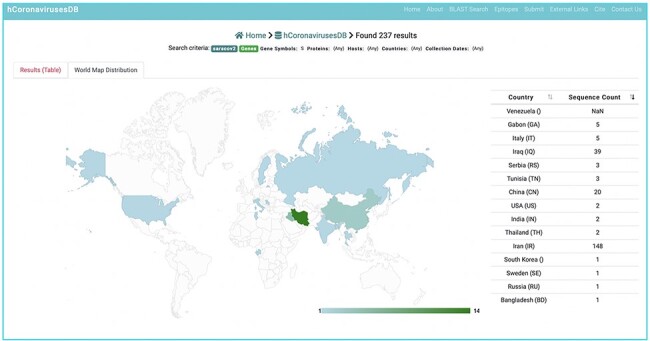
Customized BLST search of the hCoronavirusesDB. BLAST search query interface and BLAST.

To further enhance sequence analysis, the distribution of the collected sequence data can be visualized according to country of origin using the Geo Map feature from Google Chart (http://developers.google.com/chart/). Geo Map is built in HTML5/SVG, and the colour intensity for a given geographical location illustrates the number of collected sequences from that location (Figure 7).

### Database interface design

A user-friendly interface was designed for hCoronavirusesDB to facilitate the search, retrieval, and analysis of nucleotide and protein sequences associated with SARS-CoV, MERS-CoV, and SARS-CoV-2. Vue.js was applied (https://vuejs.org/) as a front-end JavaScript framework for building the hCoronavirusesDB user interface. The user can explore the catalogue of gene and protein sequences by searching for a specific accession number or by navigating through the customized search interface. In the search interface, the user can query sequences by selecting the virus name, the sequence type (gene or protein), and one or more of the following annotation categories: product name (gene symbol), host species, country, and collection year. The user can then view the identified records, including annotation details; download the sequencing data records in FASTA format; and perform multiple sequence alignments on the desired records. The communication between the hCoronavirusesDB front-end and back-end servers utilizes JavaScript AJAX calls. The source codes for the front-end and back-end hCoronavirusesDB frameworks are available at GitHub, at https://github.com/imane-boudell/vuehcovdb and https://github.com/imane-boudell/pyhcovdb, respectively, under an open-source Apache License (Version 2.0). The back-end and front-end frameworks were adopted from the Measles, Mumps, and Rubella Viruses Database (MMRdb) (20).

## Discussion

The race to develop effective vaccine candidates against SARS-CoV-2 started soon after the first SARS-CoV-2 sequence was published. Currently, over 180 vaccine candidates are being tested in various preclinical and clinical studies, and several vaccines have been granted full approval or have been approved for emergency use (21–26). Despite the unprecedented efforts to develop vaccines against SARS-CoV-2, obstacles to equitable vaccine distribution worldwide remain, and the continuous circulation of SARS-CoV-2 at an escalating rate has resulted in the continued emergence of variant SARS-CoV-2 strains. New variants have become the predominant strains in some countries, making an integrated resource that contains genomics, proteomics, and immunomic data collected for the virus a critically important development. The availability of integrated, in-house tools will facilitate the monitoring of the mutational landscape and geographic spread of SARS-CoV-2 variants around the globe. Furthermore, hCoronavirusesDB is unique in the integration of bioinformatics data for all highly pathogenic human CoVs, across various host species and geographic locations. Despite the availability of high-quality, experimentally verified epitope data, and the high degree of sequence similarity between SARS-CoV-2 and SARS-CoV and the relative similarity between SARS-CoV-2 and MERS-CoV, several fundamental issues associated with antigenic cross-reactivity and pattern recognition among human CoVs remained unexplored. hCoronavirusesDB aims to bridge this gap by improving the accessibility of these data and integrating them into a single resource. hCoronavirusesDB will continue to serve as a valuable tool that integrates genomics, proteomics, and epitope data for highly pathogenic human CoVs as they become available.

## Supplementary Material

baac017_Supp
