# Supplementary figures and images for: hCoronavirusesDB: an integrated bioinformatics resource for human coronaviruses
Source: Database (Oxford). 2022 Mar 26;2022:baac017. doi: 10.1093/database/baac017 (PMC9216473; doi:10.1093/database/baac017)

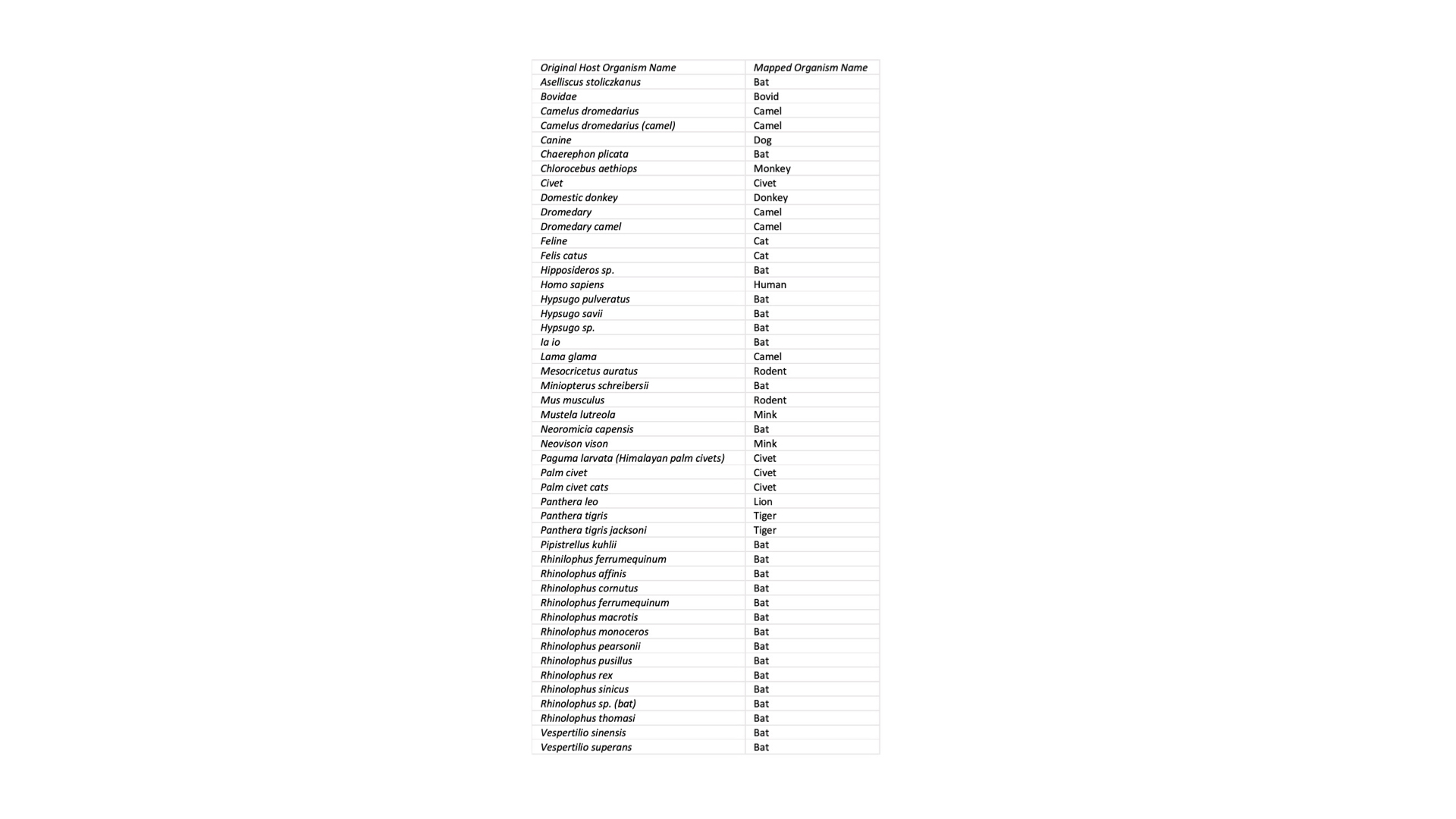

Supplement: baac017_Supp [file baac017_supp.zip › HCoronaviruses-_manuscript- Supplementary Table 1.jpeg]
